# Posttraumatic Renal Artery-Inferior Vena Cava Fistula-Induced High-Output Cardiac Failure: A Case Study

**DOI:** 10.7759/cureus.62780

**Published:** 2024-06-20

**Authors:** Raed Zughul, Cibele C Luna, Sarv Priya, Pritish Aher

**Affiliations:** 1 Radiology, University of Miami Miller School of Medicine, Jackson Memorial Hospital, Miami, USA; 2 Radiology, University of Miami, Miami, USA; 3 Radiology, University of Iowa Hospitals and Clinics, Iowa City, USA; 4 Radiology, University of Miami Miller School of Medicine, Miami, USA

**Keywords:** arteriovenous fistula, cta chest abdomen pelvis, renal artery-inferior vena cava fistula, post traumatic arteriovenous fistula, high-output cardiac failure

## Abstract

High-output cardiac failure is a less prevalent form of heart failure. Most patients with heart failure are typically categorized as having either systolic or diastolic dysfunction with elevated systemic vascular resistance. Individuals with high-output cardiac failure exhibit normal cardiac function and decreased systemic vascular resistance. This reduction may stem from diffuse arteriolar dilation or potential bypass of arterioles and capillary beds, prompting the activation of neurohormones. This case report details the diagnosis and treatment of an unusual etiology of high-output cardiac failure involving an arteriovenous fistula connecting the renal artery to the inferior vena cava and right common iliac vessels, resulting in a left-to-right shunt in a 50-year-old male patient. The report explores the etiology, pathophysiology, and clinical presentation of high-output heart failure, emphasizing the crucial role of radiology in interprofessional teams.

## Introduction

High-output cardiac failure is defined by a cardiac output that surpasses 8 L/min or a cardiac index above 4 L/min. Clinically, it manifests as increasing fatigue, swelling, and shortness of breath, both at rest and during physical activity, along with orthopnea. Some individuals may also experience chest pain and palpitations. On systemic evaluation, like other heart failure patients, those with high-output cardiac failure may present with pulmonary and peripheral edema and elevated jugular venous pressure. High-output cardiac failure typically exhibits a wide pulse pressure and warm extremities due to reduced peripheral vascular resistance. High cardiac output can result from various underlying causes, and specific symptoms and signs related to these etiologies may be evident in cases of high-output cardiac failure [1-3].

One of the etiologies of high-output cardiac failure is arteriovenous fistula (AVF). An AVF can be surgically created to provide hemodialysis access. They can also occur spontaneously or because of trauma and surgical complications, such as those following cholecystectomy and renal transplantation. AVF often results from percutaneous access of the femoral vein and artery during cardiac catheterization or after the placement of central lines in the subclavian and carotid regions. While renal AVFs are the most reported type resulting from percutaneous biopsy, they are generally self-limited and rarely require intervention. Traumatic AVFs are predominantly caused by penetrating injuries, with gunshot wounds. These fistulas can also occur due to direct arterial trauma and long bone fractures where an artery and vein are closely associated. Most traumatic AVFs are diagnosed within a week of the injury, although some can present weeks to years after the injury [4]. Sometimes, a large shunt like aortocaval fistula can manifest as a pulsatile abdominal mass or thrill, potentially mistaken for an abdominal aortic aneurysm. Lower limb weakness may also be a presenting symptom. Additionally, high-flow shunts can lead to complications like acute kidney injuries progressing to chronic kidney disease and atrophy. Endovascular repair can be a viable treatment approach for these conditions instead of open surgical repair [1,3].

## Case presentation

**Figure 1:**
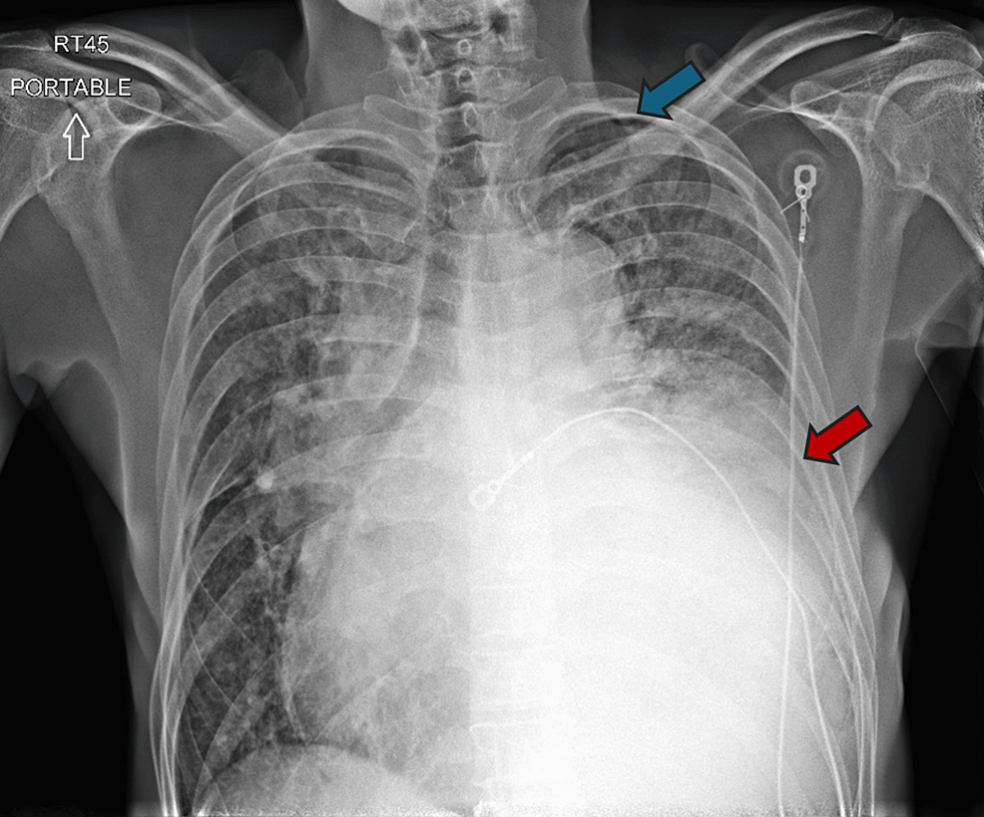
AP portable chest radiograph showing mediastinal widening, cardiomegaly, vascular congestion, pulmonary edema, left hemothorax (red arrow), and small left apical pneumothorax (blue arrow). AP, anteroposterior.

**Figure 2:**
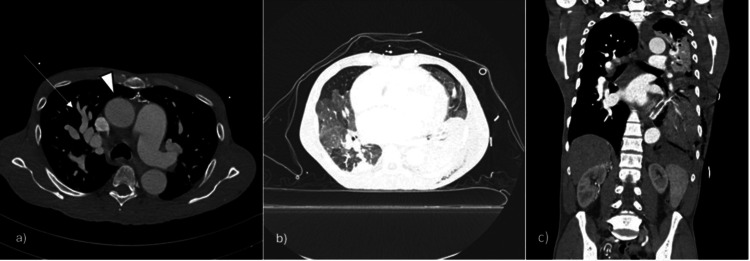
Axial images of contrast-enhanced CT scan of the chest demonstrate dilated pulmonary vessels (white arrow), normal size of the ascending aorta (white arrowhead) on the soft tissue window image (a) and pulmonary edema with left-sided hemopneumothorax on the lung widow image (b). Coronal image of contrast-enhanced CT scan of the chest and abdomen demonstrates subcutaneous edema in the left chest wall and normal right kidney size.

This case involves a 50-year-old male with a medical history of hypertension, hyperlipidemia, asthma, non-ST segment elevation myocardial infarction (NSTEMI), and a normal ejection fraction (EF) of the heart, who suffered an assault resulting in a stab wound to the right upper back and blunt injury to the lumbar region. He presented with respiratory distress, and an initial chest X-ray revealed a large hemothorax (Figure 1). A chest tube was inserted, leading to a significant bloody output and subsequent hemodynamic instability. Given the critical findings, the patient underwent an urgent posterolateral thoracotomy. The lung laceration was repaired during the procedure, and intercostal branches were ligated to control bleeding. A subsequent CT scan of the chest was done with no bleeding source detected and revealing dilated pulmonary veins (Figure 2). Endoscopy as well has failed to identify the source of the bleeding.

**Figure 3:**
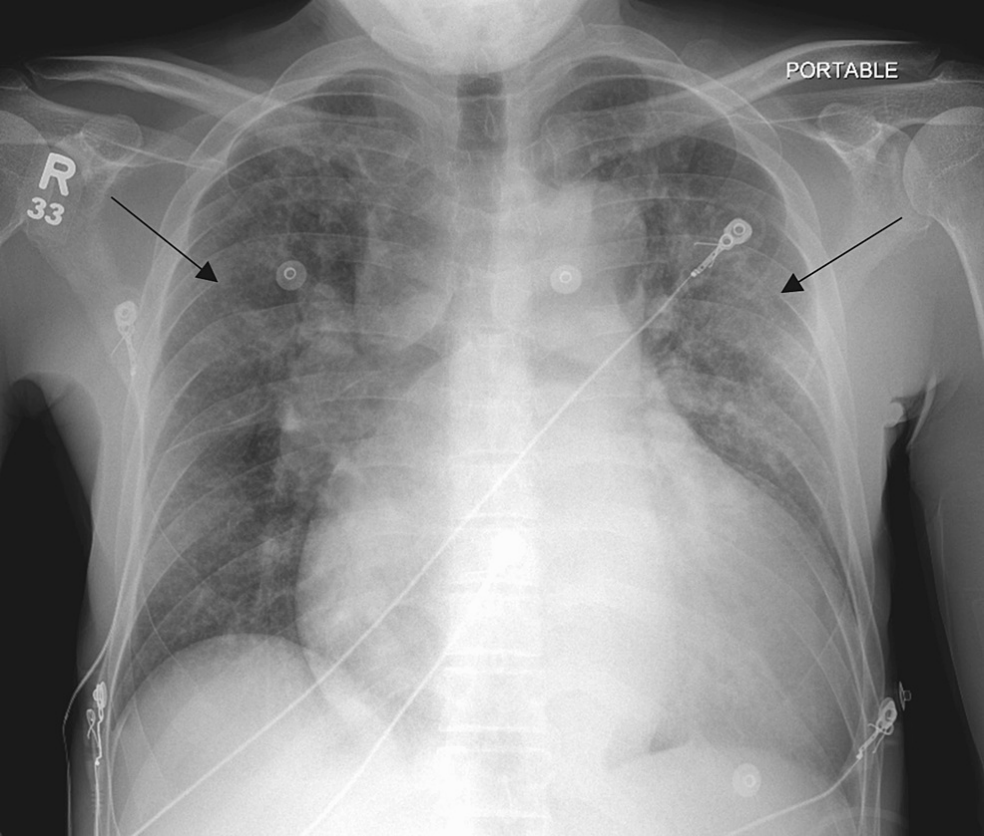
AP chest radiograph three months post-trauma showing bilateral opacities (black arrows) without evidence of pneumothorax. AP, anteroposterior.

Three months later, the patient presented to the emergency department (ED) with hypertension and tachypnea, leg swelling, distended jugular veins, and wide pulse pressure. Bedside point-of-care ultrasound (POCUS) showed dilated atria, b lines, bilateral pleural effusions, and dilated inferior vena cava (IVC) of 7 cm. His labs showed acute kidney injury, transaminitis, hyperbilirubinemia, elevated NT pro-BNP 15k pg/mL, and elevated troponins 0.098 ng/mL. He was also found to be COVID-19 positive. His chest radiograph showed bilateral opacities without evidence of pneumothorax (Figure 3). The patient was diagnosed with acute hypoxemic respiratory failure due to a hypertensive emergency with pulmonary edema and COVID-19 pneumonia, along with acute-on-chronic heart failure, and was placed on a nitro drip, Lasix, and BiPAP (bilevel positive airway pressure), and admitted to the intensive care unit. Given the dilated pulmonary veins and IVC seen on a prior CT scan (Figure 2), his pulmonary edema and congestion and hypertension were likely chronic and raised the suspicion of an AVF. A follow-up echocardiogram confirmed heart failure with borderline EF (40-45%). Cardiac catheterization was recommended; however, it was not performed due to the acute illness.

Two years later, the patient presented to the ED with mild diffuse chest pain, progressive shortness of breath, and palpitations and was found to have atrial flutter. Laboratory workup demonstrated no leukocytosis, and creatinine was elevated to 1.6 mg/dL with blood urea nitrogen (BUN) 28 mg/dL, and potassium of 4.0 mmol/L. Troponin-I elevated to 0.062 ng/mL and NT pro-BNP to 12k pg/mL. He had a further reduction in the EF (30-35%), and it was thought it might be high-output heart failure. The comprehensive metabolic panel was normal. Since the prior CT chest showed dilated IVC and plethoric lung (Figure 3), high-output cardiac failure related to AVF was suspected.

**Figure 4:**
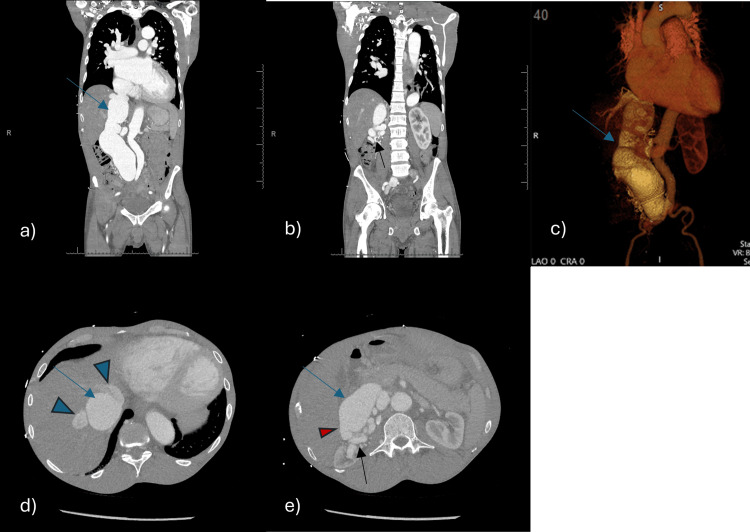
CT scan of the chest, abdomen, and pelvis coronal images (a, b), 3-D rendering reconstruction (c), and axial images (d, e) demonstrate severely dilated IVC (blue arrow in a, c, d, and e), dilated hepatic veins (arrowheads in d), tortuous and dilated vessels located over the right renal hilum (black arrow in b and e), and right renal artery-IVC fistula (red arrowhead in e). IVC, inferior vena cava.

A new CT scan of the chest, abdomen, and pelvis revealed tortuous and dilated vessels located over the right renal hilum with severely dilated IVC (Figure 4). The right kidney exhibited progressive atrophy. With the dilated renal vessels seen on the CT scan (Figure 4b, 4e), a diagnosis of AVF was suggested between the right renal artery and aorta. During angiography, a fistula between the right renal artery and IVC was identified. Additionally, another fistula involving the common iliac vessels was diagnosed. Treatment options, including open surgical procedures and endovascular repair, were discussed. Endovascular solutions were limited due to the specific anatomy, while open procedures posed a significant risk of bleeding. Two weeks later, the patient underwent angiography, intending to conduct an endovascular repair. Contrary to the expectation of finding an IVC-aorta fistula, the angiography revealed a fistula between the right renal artery and IVC, and another fistula involving the right common iliac artery and vein. Ultrasound (US)-guided access to the right common femoral artery was obtained, followed by stenting of the right common iliac artery. Additionally, a 20 mm Amplatzer occluder device was placed in the right renal artery to address the AVF.

**Figure 5:**
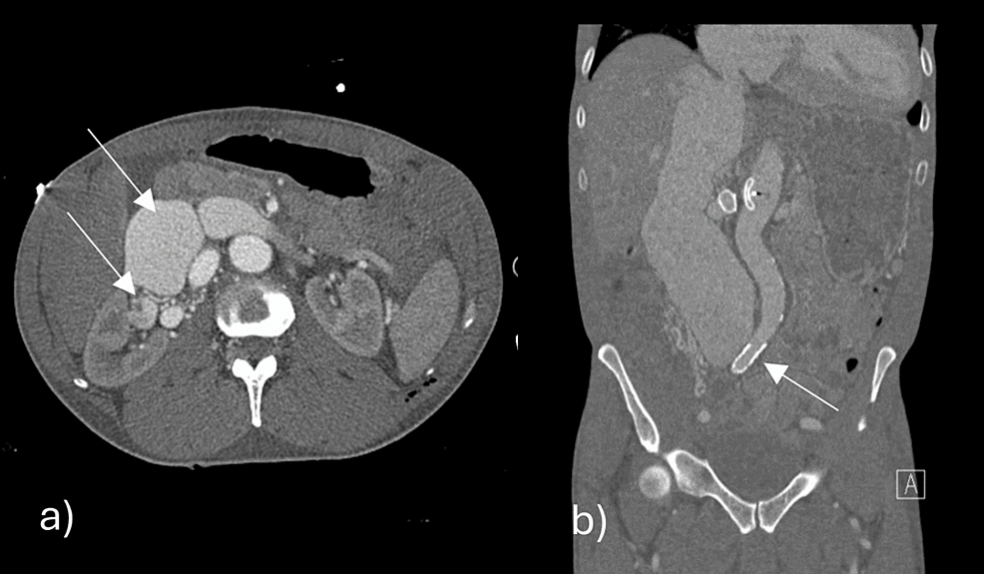
CT angiography of the chest, abdomen, and pelvis axial image (a) and coronal image (b) demonstrate a reduction in the size of dilated and tortuous renal vessels and inferior vena cava (arrows in a), alongside a patent stent in the right common iliac artery (arrow in b).

One-month post-intervention, a CT angiography of the chest, abdomen, and pelvis revealed a reduction in the size of dilated and tortuous renal vessels and IVC, alongside a patent stent in the right common iliac artery (Figure 5).

## Discussion

Heart failure is a prevalent condition, leading to millions of office visits annually and standing as the most frequent diagnosis among hospitalized patients in the United States alone. Heart failure is a significant health concern in the United States, affecting approximately 6.2 million adults. In 2018, it accounted for 13.4% of all deaths. Over 500,000 new cases are diagnosed each year, with a current prevalence estimated at around 5 million. This number encompasses all types of heart failure, including systolic and diastolic dysfunction, as well as high-output cardiac failure. However, isolated high-output cardiac failure is relatively rare, and its exact incidence and prevalence remain unknown, possibly because high-output cardiac failure often arises secondary to other underlying conditions, making its identification less straightforward [5,6].

High-output heart failure can arise from various causes, which can be congenital, acquired, or iatrogenic. Some of the common causes include hyperthyroidism, myeloproliferative disorders, sepsis, beriberi, chronic lung disease, AVFs, liver cirrhosis, obesity, severe chronic anemia, Paget’s disease, and carcinoid syndrome. Among these, AVFs are a rare but potential cause of high-output heart failure [5-7].

The primary pathophysiology underlying high-output heart failure involves a decrease in systemic vascular resistance, which can stem from either peripheral arterial vasodilation or arteriovenous shunting. This phenomenon can occur through various mechanisms, such as the inflammatory response inducing peripheral vasodilation (as observed in sepsis), cellular products being released in response to hormones, or the direct bypassing of the capillary bed, leading to increased venous return to the heart and subsequently elevated cardiac output (as seen in shunting due to AVFs). However, these mechanisms share common features, including decreased systemic vascular resistance, a reduction in the arteriovenous oxygen gradient, and an increase in cardiac output. Despite the elevated cardiac output, it remains insufficient to meet the body's demands, ultimately resulting in clinical heart failure. This inadequate blood supply triggers neurohormonal responses akin to other forms of heart failure, such as the activation of the renin-angiotensin-aldosterone system (RAAS), the adrenergic nervous system, and the excessive release of antidiuretic hormone [1,5-7]. The etiology of high-output heart failure should first be evaluated using clinical history and physical examination.

Then comes the role of radiology, including chest radiographs and CT scans, which can show cardiomegaly, pulmonary congestion or venous dilation, pleural effusion, pulmonary edema, and pneumonia. Following these investigations with echocardiography, when heart failure is suspected, is essential. In high-cardiac-output states, echocardiography may reveal a preserved left ventricular EF. It is important to note that high-output heart failure can occur even when left ventricular systolic function appears normal. Over time, patients may develop compensatory left ventricular dilatation with or without hypertrophy. These changes can lead to worsening heart failure, which can be followed by laboratory investigations. Such laboratory workup includes mixed venous oxygen saturation through venous blood gas assessment and natriuretic peptide level evaluation elevated in heart failure. Other laboratory tests like thyroid function tests, liver function tests, and serum electrolyte levels help identify the specific cause of high-output cardiac failure and guide appropriate treatment and management strategies. Although angiography may be warranted, a contrast-enhanced CT scan remains the gold standard for diagnosing and identifying AVFs [2].

US Doppler is a non-invasive and cost-effective method to confirm the diagnosis of AVFs, particularly when the AVF is peripheral. The duplex US typically reveals low-resistance flow in the feeding artery. High-velocity flow and turbulence are evident at the anastomosis site or fistula. Additionally, vessel dilation, thickened walls, and high-velocity flow patterns are present in the draining veins or venous plexus associated with the AVF. Finally, conventional angiography is also helpful in detecting the exact location and for surgical planning with endovascular stents.

In our case of high-output cardiac failure, a history of trauma was accompanied by clinical signs and symptoms suggestive of cardiac failure despite normal cardiac output. Elevated laboratory markers further supported the diagnosis. Additionally, a CTA of the chest, abdomen, and pelvis revealed the presence of an AVF between the right renal artery and the IVC. Radiology played a crucial role in both diagnosing and managing the condition, particularly in addressing the AVF involving these vascular structures.

The peripheral AVFs can be corrected using an endovascular stent. Similarly, aortocaval fistulas are also addressed with endovascular stents, mainly due to the elevated mortality risk associated with open surgical procedures [3,8].

## Conclusions

High-output cardiac failure can arise from congenital, acquired, or iatrogenic causes. Arteriovenous fistulae are a rare cause of high-output heart failure involving a decrease in systemic vascular resistance from peripheral vasodilation or arteriovenous shunting. Radiology plays an important role in diagnosing and managing high-output cardiac failure and potential causes such as AVF. Such fistulae can be corrected through endovascular stenting given the elevated mortality risk associated with open surgical procedures.

## References

[1] Charif F, Nassar P, Youssef D, Neghawi Z, Saab M (2021). High output heart failure secondary to aorto-caval fistula treated with an Amplatzer septal occluder: Case report and review of literature. Cureus.

[2] Tonolini M, Ippolito S, Rigiroli F (2014). Images in medicine: Spontaneous aortocaval fistula complicating abdominal aortic aneurysm. J Emerg Trauma Shock.

[3] Almumtin A, Dahman M, Khalil B, Alabduljabbar M, Almusahel E, Koussayer S (2023). Renal-inferior vena cava fistula complicating laparoscopic cholecystectomy causing heart failure repaired endovascularly, a case report and literature review. Int J Surg Case Rep.

[4] Şahin M, Yücel C, Kanber EM, İlal Mert FT, Bıçakhan B (2018). Management of traumatic arteriovenous fistulas: A tertiary academic center experience. Ulus Travma Acil Cerrahi Derg.

[5] Chang RS, Hu JR, Beckman JA, Forbes RC, Shawar SH, Concepcion BP (2021). High output heart failure associated with arteriovenous fistula in the setting of kidney transplantation. Kidney Int Rep.

[6] Mehta PA, Dubrey SW (2009). High output heart failure. QJM.

[7] Singh S, Sharma S (2023). High-output cardiac failure. StatPearls [Internet].

[8] Saleh MA, El Kilany WM, Keddis VW, El Said TW (2018). Effect of high flow arteriovenous fistula on cardiac function in hemodialysis patients. Egypt Heart J.

